# Supplementary figures and images for: Intranasal challenge with B. pertussis leads to more severe disease manifestations in mice than aerosol challenge
Source: PLoS One. 2023 Nov 2;18(11):e0286925. doi: 10.1371/journal.pone.0286925 (PMC10621807; doi:10.1371/journal.pone.0286925)

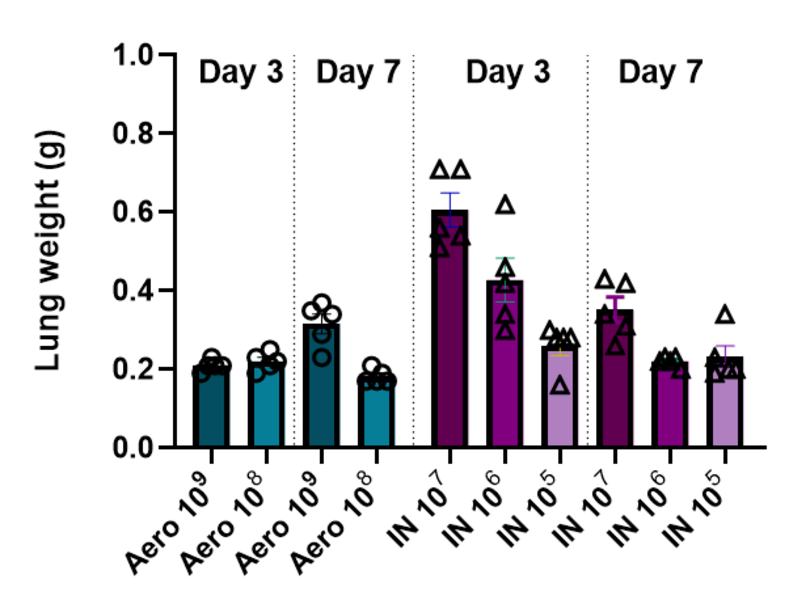

Supplement: S1 Fig — Error bars are mean ± SEM values (n = 5 per group). ANOVA with Kruskal Wallis comparisons were performed for the high dose (Aerosol 109 vs IN 106) and no statistical differences were found between these groups at the timepoints depicted in this figure. (TIF) [file pone.0286925.s001.tif]

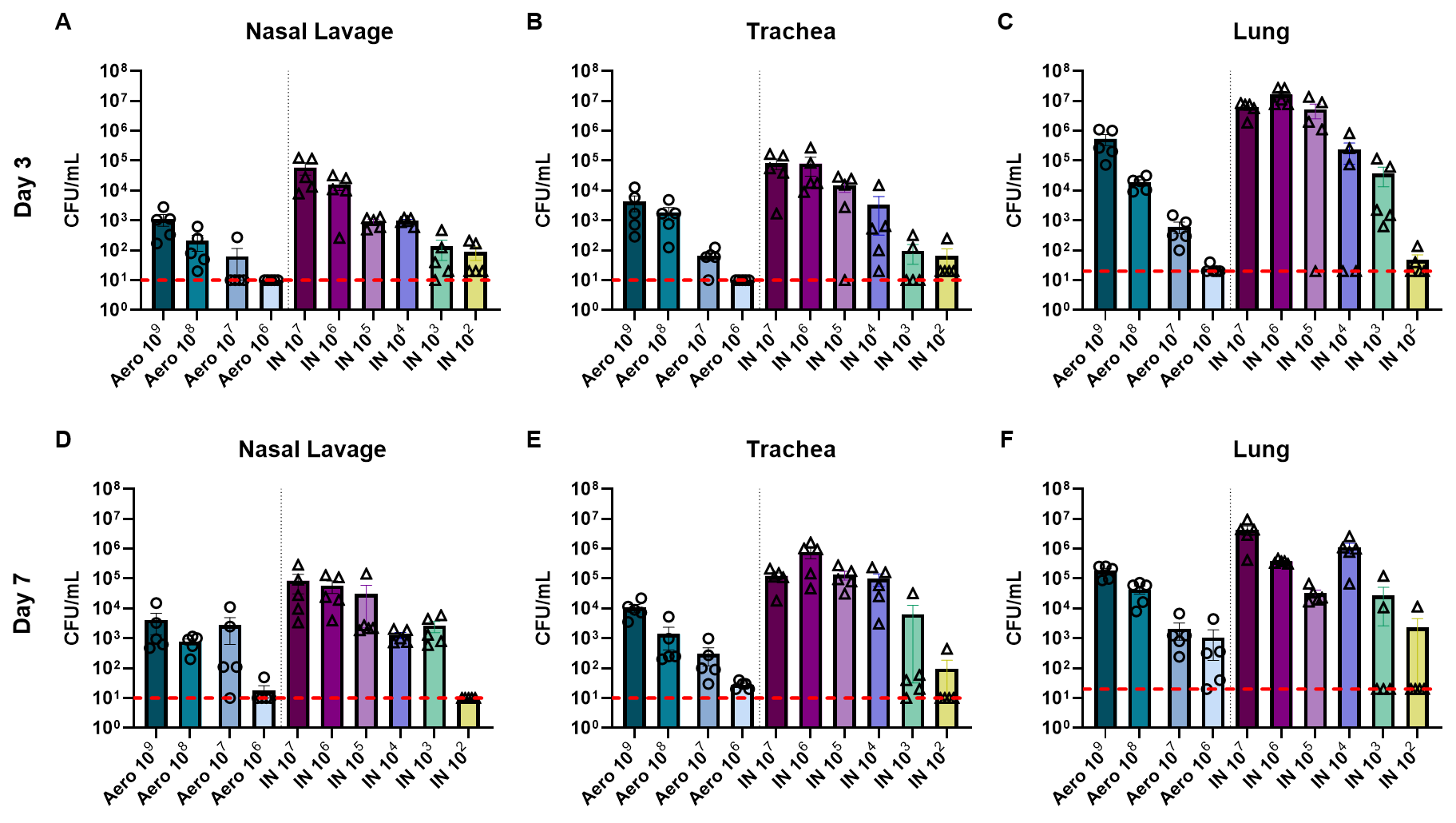

Supplement: S2 Fig — Bacterial burden in the A) nasal lavage, B) trachea, and C) lung of mice at day 3 post-challenge. Bacterial burden in the D) nasal lavage, E) trachea, and F) lung of mice at day 7 post-challenge. Error bars are mean ± SEM values (n = 5 per group). (TIF) [file pone.0286925.s002.tif]

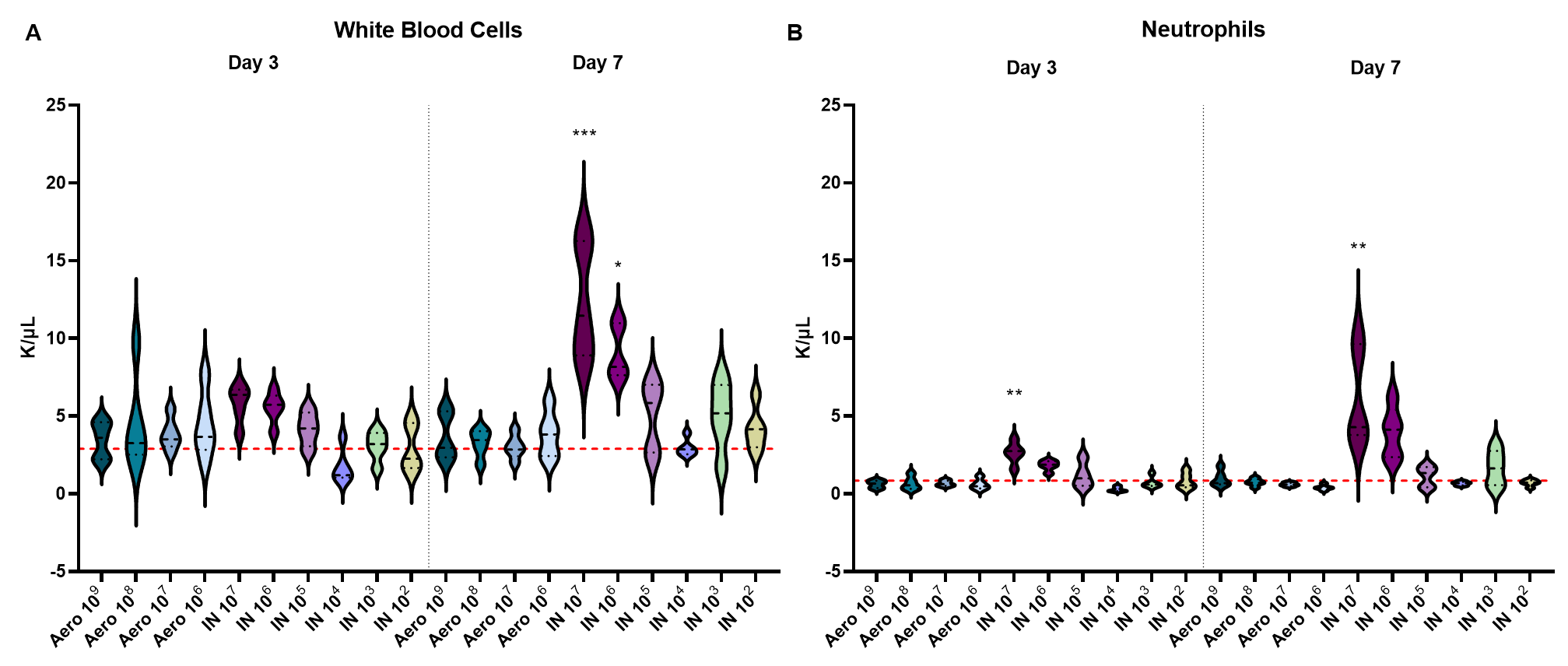

Supplement: S3 Fig — A) White blood cell counts and B) neutrophils measured at day 3 and day 7 post-challenge. Stars represent comparison to baseline. The p-values were calculated using ANOVA followed by a Tukey’s multiple-comparison test, * p < 0.05, ** p < 0.01, and *** p < 0.001 (n = 5 per group). Error bars are mean ± SEM values. (TIF) [file pone.0286925.s003.tif]

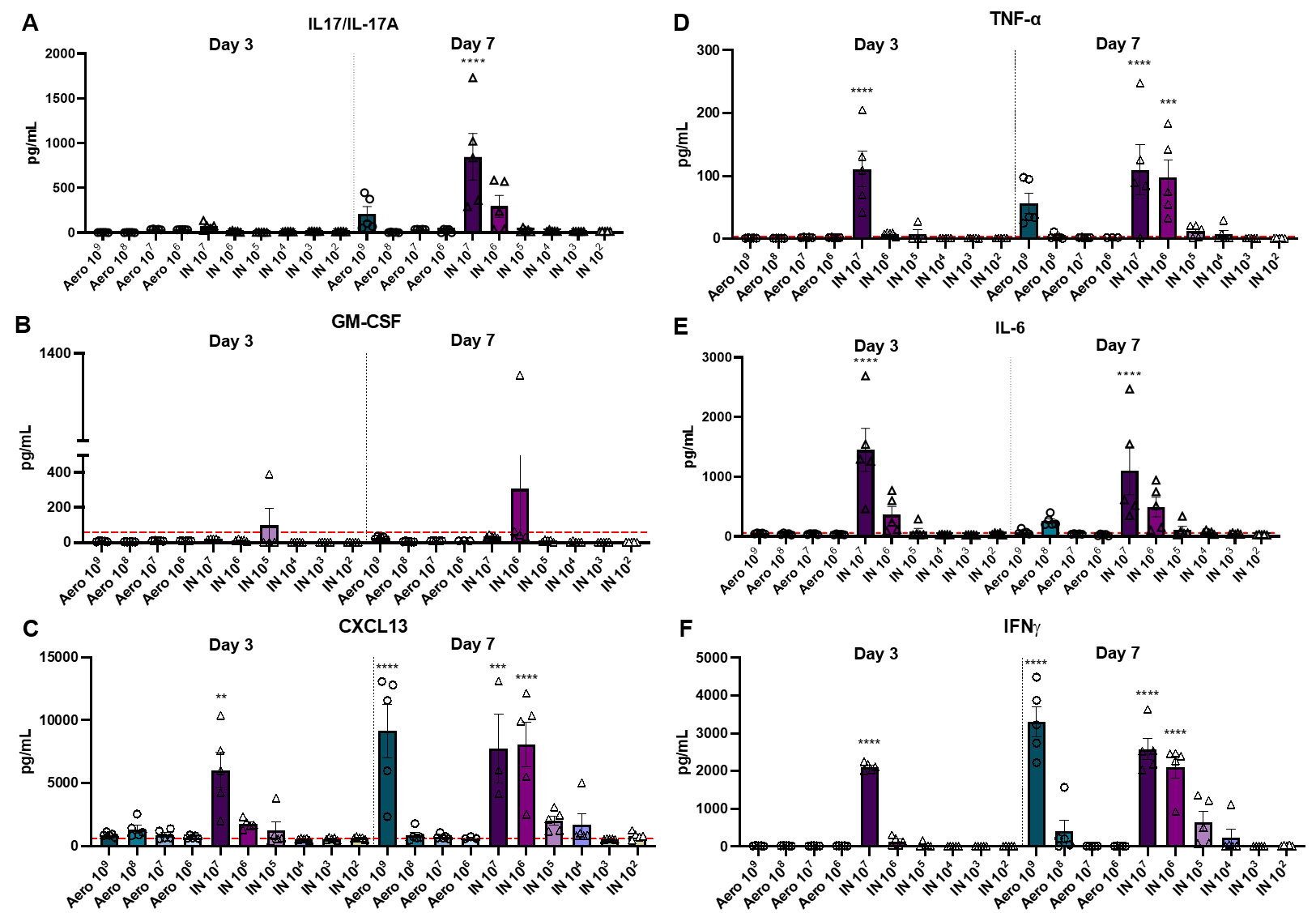

Supplement: S4 Fig — A) IL-17/IL-17A B) GM-CSF, C) CXCL13, D) TNF-⍺, E) IL-6, and F) IFN-γ represented as pg/mL for all challenge doses. The p-values were calculated using ANOVA followed by a Tukey’s multiple-comparison test, ** p < 0.01, *** p < 0.001, and **** p < 0.0001 (n = 5 per group). Error bars are mean ± SEM values. (TIF) [file pone.0286925.s004.tif]

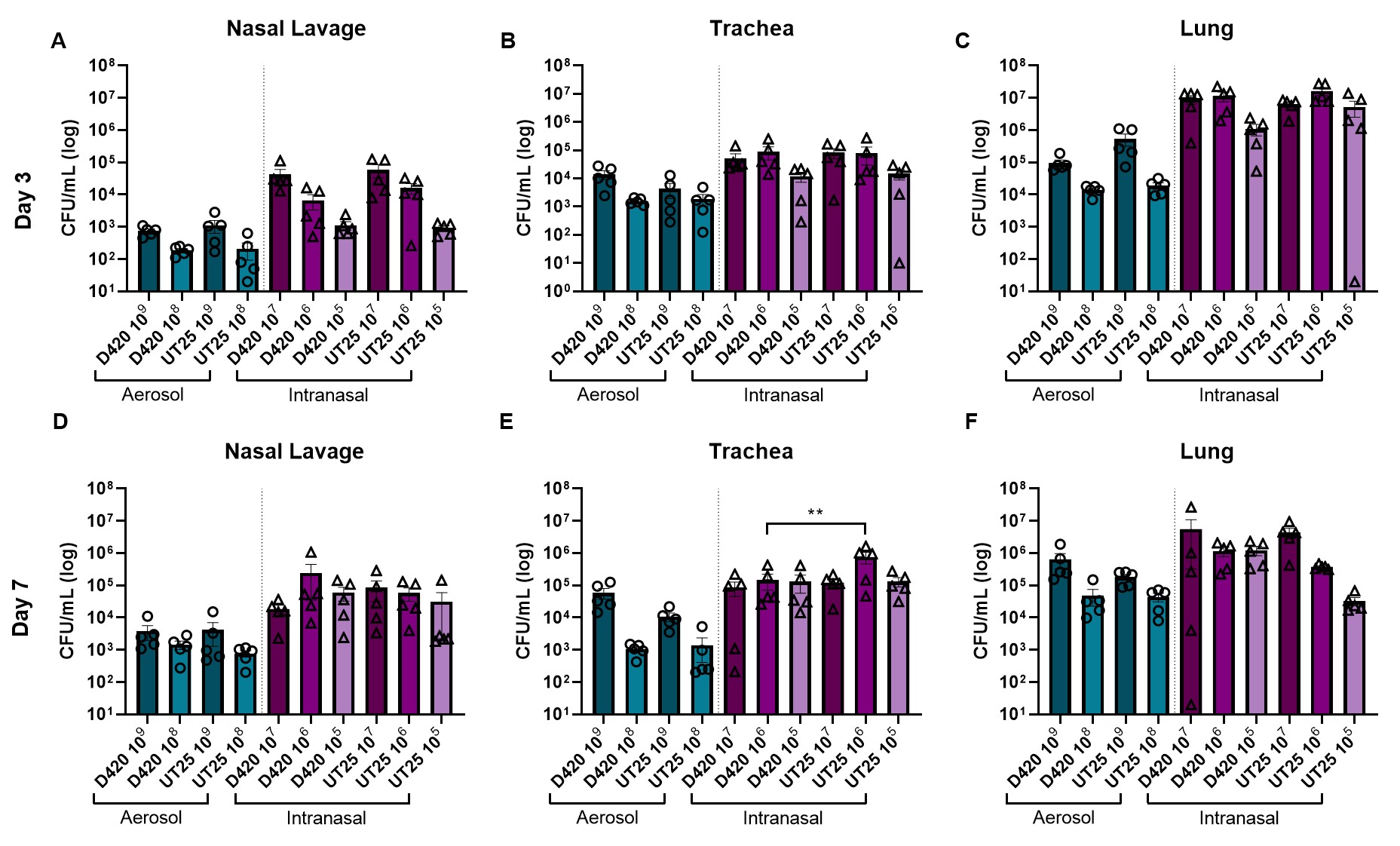

Supplement: S5 Fig — Bacterial burden in the A) nasal lavage, B) trachea, and C) lung of mice at day 3 post-challenge. Bacterial burden in the D) nasal lavage, E) trachea, and F) lung of mice at day 7 post-challenge. Error bars are mean ± SEM values (n = 5 per group). (TIF) [file pone.0286925.s005.tif]

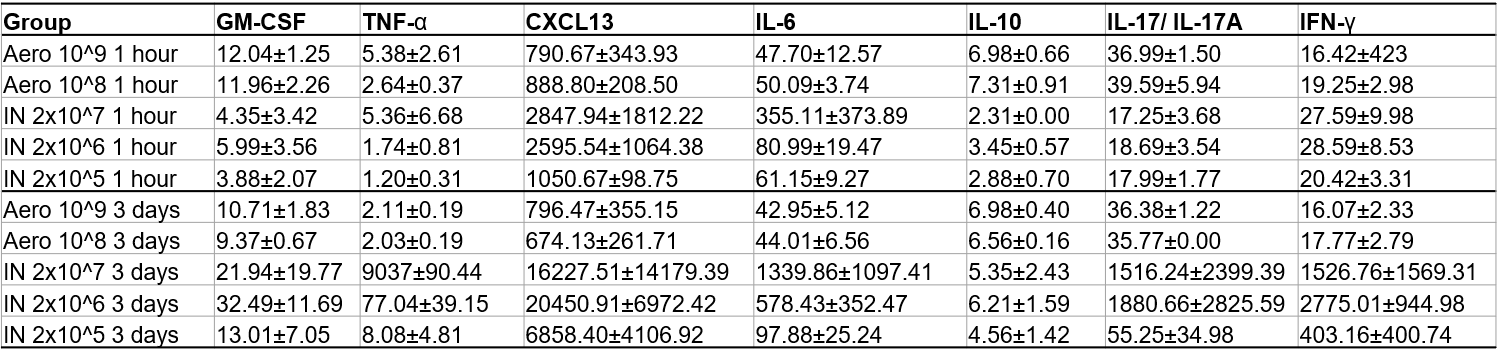

Supplement: S6 Fig — Data are represented as mean ± SEM values in pg/mL. (TIF) [file pone.0286925.s006.tif]
